# Nudging in the nursing home: A qualitative interpretive study

**DOI:** 10.1016/j.ijnsa.2024.100287

**Published:** 2024-12-29

**Authors:** Anne Helene Mortensen, Dagfinn Nåden, Dag Karterud, Vibeke Lohne

**Affiliations:** Faculty of Health Sciences, Department of Nursing and Health Promotion, OsloMet–Oslo Metropolitan University, Oslo, Norway

**Keywords:** Care Routines, Experienced caregivers, Nudging, Nursing homes, Soft paternalism, Dignity, Autonomy

## Abstract

**Background:**

Nudging involves deliberately changing the environment or context to induce better choices. Several studies consider such methods unethical manipulation that threatens the principles of informed consent and autonomy, which are particularly vital in healthcare. Others argue that nudging respects personal freedom because it is not in conflict with the person's explicit values or choices, beneficial, and easy to resist. Additionally, studies argue that such strategies are legitimate as they can prevent more intrusive forms of paternalism. Given the increasing prevalence of soft paternalism through nudging, there is a need to examine its use in healthcare.

**Objective:**

This study explored the phenomenon of nudging in a nursing home context.

**Design:**

A qualitative interpretive design informed by Gadamer's hermeneutics was employed.

**Setting:**

Three distinct nursing home units in Norway, including long-term, dementia, and rehabilitation units. The study was conducted between December 7^th^. 2019 and July 2^nd^. 2020.

**Participants:**

Individuals involved in the units during the observation period.

**Methods:**

Seven to eight weeks of observations, followed by interviews with caregivers (nurses and auxiliary nurses), two occupational therapists, and two physiotherapists.

**Results:**

The results suggest that nudging in this context can be understood across three themes: modification of physical surroundings, information and interaction, and ambiguous interventions.

**Conclusions:**

Nudging is being used in nursing homes to guide residents’ choices and behaviors to foster their well-being and uphold social norms. However, caregivers are also navigating the use of diverse forms of influence, including subtle nudging and assertive persuasion, and must consider complex factors.

**Registration number:**

REK – Regional ethics committee case number 175774

Sikt - Norwegian Agency for Shared Services in Education and Research case number 248550.

**Social media abstract:**

Experienced caregivers in nursing homes effectively use nudging to guide care routines, resulting in efficient care and better interactions.

**What is already known**

 

• Nudging involves deliberate changes in the environment or context of decision-making to induce better choices and is an increasingly prevalent practice.
• Nudging has raised ethical concerns of perceived manipulation that can infringe upon the principles of informed consent and autonomy, which are especially crucial to healthcare settings.

Alt-text: Unlabelled box

**What this paper adds**

 

• This study shows that experienced caregivers effectively use nudging to guide care routines, resulting in efficient care and better interactions.
• This study found that an excessive focus on residents’ wishes can sometimes negatively impact caregiver-resident interaction, and shows the complex considerations caregivers navigate when exerting influence, including subtle nudging and assertive coercion.
• This study acknowledges the ethical concerns regarding nudging potentially infringing on residents’ autonomy in a nursing home context and considers how it may protect and support their dignity.

Alt-text: Unlabelled box

## 1 Background

Traditionally, healthcare services have been marked by a paternalistic approach in which health professionals wield more power than residents (Genuis, 2021). However, there has been a shift in the key ethical values in nursing homes towards enhancing residents' autonomy and promoting dignity (Moilanen et al., 2021; Moilanen et al., 2022). This shift has given rise to dilemmas regarding balancing resident autonomy with the responsibility of beneficence (Cheraghi et al., 2023; Jacobson and Silva, 2010) as caregivers strive to respect residents' rights to make their own decisions while also ensuring their well-being.

Alongside this shift, the use of choice- and behavior-modifying strategies, such as nudging, the practice of altering the choice context to influence choice outcome (Thaler and Sunstein, 2008) has increased. These strategies are often seen as paternalistic and thus appear to contradict the emphasis on residents' autonomy (Abdukadirov, 2016; Holm, 2019). Along these lines, some view nudging as manipulative and ethically challenging (Holm, 2019), while others see it as a helpful and benevolent tool that can enhance autonomy (Engelen, 2019; Sunstein, 2015). Considering this, nudging's role in healthcare and its ethical implications are controversial.

Despite this controversy, the practice of shaping the environment to influence choice outcomes is common in many health care settings (Hanna and Grill, 2018). In healthcare, the goal of nudging has typically been to facilitate decision-making, enhance patient outcomes, and contribute to more efficient healthcare delivery (Kuyer and Gordijn, 2023; Mortensen et al., 2019). However, the meaning and implications of nudging in a clinical context require further exploration. This paper reports the first study exploring the use of nudging within a healthcare context.

As briefly outlined above, nudging refers to deliberate changes in the environment or context wherein efforts are made to induce better choices (Thaler and Sunstein, 2008). This can change residents’ choices and behavior through small changes to the environment where the choice is made or to the presentation of the choice. This typically occurs by activation of our cognitive biases (Thaler and Sunstein, 2008) that control our intuitive decision-making (Kahneman, 2011) without activating deliberations.

Presentation, timing, and framing of information influence the decision-making process and, consequently, the choice made (Thaler and Sunstein, 2008). For example, providing information on survival rates rather than mortality rates influences subsequent treatment decisions (Redelmeier et al., 1993). Common examples of such nudging techniques are framing and default choices (Thaler and Sunstein, 2008). Not all influences on choices are categorized as nudging. The criterion for an influence to be called a “nudge” is that it must 1) precede the decision, 2) benefit the one who is influenced, and 3) be easy to resist (Thaler and Sunstein, 2008).

Several studies consider the act of influencing people's decisions through nudging unethical manipulation, threatening the principles of informed consent and autonomy, which are crucial in healthcare settings (Holm, 2019; Ploug, 2019). Conversely, others argue that nudging respects a person's freedom because it does not conflict with their explicit values or choices, benefits the person, and is easy to resist (Clavien, 2018; Engelen, 2019). Key questions include who decides if a nudge benefits the individual and if a nudge is easy to resist.

Previous research justifies nudging by suggesting it can prevent more intrusive use of paternalism (Mortensen et al., 2019; Saghai, 2013). Despite this ongoing discussion, nudging is becoming an increasingly prevalent practice in our society, possibly at the expense of autonomy. Hence, what nudging entails in a clinical context and how it influences residents’ dignity requires further exploration.

The concept of autonomy refers to the human right to self-govern, the right to make individual decisions, decide our own values and ends, and pursue them (Beauchamp, 2019; Killmister, 2018). It also identifies conditions to determine if a choice is autonomous or not. An autonomous choice must be motivated by second-order values and desires, cannot be a product of manipulation or oppression, and must be justifiable by the one choosing in hindsight (Killmister, 2018). Sometimes the concept of autonomy refers to a person's capacity to make autonomous decisions, implying the capability for rationality and critical reflection (Killmister, 2018). Traditional definitions of autonomy emphasize independence, self-sufficiency, and the ability to reason (Genuis, 2021).

Paternalism is defined as disregarding a resident's autonomy to protect or benefit the resident. Therefore, whenever autonomy and beneficence are discussed, paternalism is the key tension (Beauchamp, 2019; Jacobson and Silva, 2010). Acting against a person's explicit wishes is typically considered as hard paternalism (Killmister, 2018). Conversely, when the choice is not actually autonomous, or the resident's capacity for autonomy is compromised, it is typically considered soft paternalism. Soft paternalism aims to align a person's choices and actions with their actual goals and values, thus presupposing a mismatch between the expressed and the authentic will (Killmister, 2018)*.*

Nudging is often deemed paternalistic because the person responsible for the nudging influences someone else's choices for their benefit. Advocates of nudging differentiate between soft and hard paternalism based on how easy it is to resist the influence. As such, a nudge, which can be easily resisted, is considered soft paternalism (Thaler and Sunstein, 2008), while pressure and coercion, which are harder to resist, are deemed hard paternalism.

Manipulation is defined as any action other than rational persuasion intended to influence residents’ behavior in a specific direction and performed such that they consider their new behavior as a free choice (Cohen, 2013). The immorality of manipulation stems from the disconnection of choices and behaviors from their guiding reasons, thus making them normatively flawed (Gorin, 2018). Typically, manipulation involves misleading an individual's beliefs, desires, or emotions such that they fail to follow their reasons or intent. This typically implies that the manipulated individual is being used as a means to someone else's ends (Barnhill, 2014; Gorin, 2018). However, paternalistic manipulation is when a resident is manipulated to protect or benefit the resident (Gorin, 2018).

The concept of dignity is closely linked to autonomy (Gallagher, 2004; Jacobson, 2009; Killmister, 2020), and preserving dignity is a primary ethical goal in health services and policy. In this study we understood dignity as comprising three intertwined strands of personal, social, and status dignity (Killmister, 2020). Status dignity refers to others treating us as appropriate to our status or position (human being, doctor, resident, and so on), while personal and social dignity refers to the upholding or transgression of the norms applicable to us (Killmister, 2020).

Broadly, as noted above, this study aimed to contribute to the literature by exploring the concept and phenomenon of nudging within the context of nursing homes.

## 2 Methods

### 2.1 Design

This study adopted a qualitative design informed by Gadamer's hermeneutics to explore the concept and phenomenon of nudging in relation to nursing homes. Fundamental elements of this approach include the hermeneutical circle, the historicity and contextuality of truth, and the fusion of horizons (Gadamer, 2013). Aligned with these elements, this study emphasized the importance of the continuous awareness of our own preconceptions, recognizing these as essential to interpretations and understanding (Gadamer, 2013). Preconceptions in this study were mainly influenced by caregivers experiencing dilemmas between residents’ rights to autonomy, caregivers’ duties to uphold the principles of beneficence, and the concepts of dignity, nudging, and soft paternalism (Killmister, 2018, 2020; Thaler and Sunstein, 2008).

### 2.2 Data collection

Fieldwork with participant observation (Fangen, 2010) and semi-structured interviews (Brinkmann and Kvale, 2018) were used to collect data on experiences with nudging, and how it can be understood in the nursing home context. Fieldwork provided access to firsthand experiences and a broad starting point for our interpretations (Fangen, 2010). The interviews allowed us to understand how the involved participants think and understand their actions and the situation that called for the actions (Brinkmann and Kvale, 2018).

### 2.3 Participants and context

Fieldwork (Fangen, 2010) was conducted at three different nursing homes in Norway, including a long-time unit, a dementia unit, and a rehabilitation unit by the first author (AHM). These units were purposely chosen because of their access to diverse resources, experiences with residents resisting care, and competence linked to paternalism. Additionally, these units represent the most common types of nursing home units in Norway, thus enhancing the relevance and applicability of our study within the national context. The characteristics of the participating nursing home units are presented in Table 1.

**Table 1:** Nursing home units’ characteristics.

Nursing home unit 1	The nursing home was in a rural area on the outskirts of a city divided into six units with a total of 126 residents. Five units were traditional long-term units, and one unit was a dementia unit. The participating unit was a long-term unit with 30 residents.
Nursing home unit 2	The nursing home was a municipal long-term home for people with dementia located in a city, and had 55 residents. It was divided into two sections, with four housing units in each section. The participating unit was a housing unit with eight residents.
Nursing home unit 3	The nursing home was in a rural town and had 86 residents divided into two units with long-term residents and two units with intermittent placements, rehabilitation placements, and short-term placements. The participating unit had 27 residents at the time of the observations. Residents in this unit stayed for a limited period. Some had intermittent stays between home care and the nursing home, some stayed for rehabilitation after being hospitalized, and some were there to decide on the proper care level and future placement in a long-term unit.

Nearing the end of each observation period caregivers participated in semi-structured qualitative interviews (Brinkmann and Kvale, 2018) with the researcher. The inclusion criteria for the interview were being employed in a participating nursing home unit and involved in the residents’ daily care. The sample of 20 interview participants was purposely chosen, striving for a balanced representation of gender and professions involved in daily resident care. In qualitative research, smaller sample sizes are preferred to support the depth of understanding. At the same time, it is important to include enough participants to achieve saturation (Creswell and Creswell, 2018). We aimed to achieve this through purposeful sampling, seeking to cover various perspectives. The characteristics of the interview participants are presented in Table 2, Table 3.

**Table 2:** Interview participants’ professions.

Profession	Total	Nursing home unit 1	Nursing home unit 2	Nursing home unit 3
Nurses	**5**	1	1	3
Auxiliary Nurses	**11**	4	4	3
Occupational Therapists	**2**	0	0	2
Physiotherapists	**2**	0	0	2
**Total**	**20**	**5**	**5**	**10**

**Table 3:** Interview participants’ gender, age, and experience with resident/patient group.

Gender, age, and experience	Total	Nursing home unit 1	Nursing home unit 2	Nursing home unit 3
Male	5	1	3	1
Female	15	4	2	9
Age		23-41 years (mean 31)	46-53 years (mean 49,5)	24-50 years (mean 39)
Experience		1-12 years (mean 5)	10-29 years (mean 18)	0,5-18 years (mean 12)

Consistent with the gender imbalance in nursing homes, only five of the participating caregivers were men.

#### 2.3.1 Participant observations

The first author performed eight weeks of participant observation (Fangen, 2010) at each nursing home unit. The various observation sessions lasted from 3.5 h to 7 h. During the observations, the researcher strived for a balance between active participation and distant observation. The closeness of active participation enabled researchers to get close to the participants to understand them, but a distance was necessary to be able to observe interactions from the outside (Fangen, 2010). Field notes with thick descriptions (Fangen, 2010) were written in Norwegian, daily after participant observation, wherein the researcher extensively noted the day's observations, personal reactions, reflections, and preliminary interpretations. The field notes comprised more than 150 pages of text. All observations are theory-laden (Hanson, 1958), and it is unrealistic to interpret or understand observation results without preconceptions (Gadamer, 2013). Hence, this study's aim and theoretical framework influenced the observations and the descriptions in the field notes.

#### 2.3.2 Interviews

Participating caregivers became familiar with the researcher during the participant observation period that preceded the interviews. The participants were informed of the researcher's background as a university lecturer in nursing, and the study's purposes. All interviews were conducted in Norwegian in a private small meeting room close to the unit. The caregivers were encouraged to describe their work aims and the situations they experienced as challenging or successful. They were also asked about their experiences with motivating residents and those refusing care. Follow-up questions and silence were used to stimulate participants to deepen their descriptions. Toward the end of the interviews, follow-up questions and paraphrasing were used to verify the interviewer's interpretations (Brinkmann and Kvale, 2018). The interviews were concluded with a question regarding any other things the participants wanted to share. Field notes were written in Norwegian immediately after each interview. Audio recordings of the interviews were uploaded to a secure platform and transcribed verbatim by the interviewer AHM.

### 2.4 Analysis and interpretation

The study's aim and the preconceptions in our horizon of understanding were crucial for interpretation (Gadamer, 2013) throughout this study. Preconceptions and aims influence what a researcher sees and hears (Brinkmann and Kvale, 2018; Gadamer, 2013; Hanson, 1958). This means that our initial understanding influenced what the researcher noticed during the observations, noted in field notes, and inquired in the follow-up questions during observations and interviews. Without a preconception of the concept of nudging, the researcher could not have observed nudging in clinical practice. Clarifying and structuring the field notes and transcription of interviews also involved a new interpretation of the data material (Brinkmann and Kvale, 2018; Fangen, 2010). After these initial interpretation steps, the first author started the formal analysis and interpretation through an iterative process of reading and interpreting the texts. The first step was an open reading of all material (Brinkmann and Kvale, 2018). In this phase, the field notes and transcriptions of the interviews were read, and memos with preliminary ideas and interpretations were written (Brinkmann and Kvale, 2018). In the next step, all text sections related to nudging were identified and coded, in addition to data sections concerning influence on residents, paternalism, autonomy, or dignity. This process of analysis and interpretation continued in an iterative movement between data excerpts, interpretations, and the entire data corpus (Gadamer, 2013). After several preliminary iterations by the first author under the supervision of the last author, the other authors were included in the process and asked to discuss the meanings of observations and quotes and validate interpretations. After this stage, the first author translated the findings, comprising excerpts of field notes and interviews, into English. Next, excerpts of field notes and interview quotes were chosen by the research group to illustrate the findings based on how well they represented the findings, all units, and variations among participants. We ensured the transparency of our interpretations by revealing the preconceptions influenced by our backgrounds and theoretical frameworks and presented our findings together with data excerpts that illustrated our interpretations. Quotes and excerpts of field notes were translated from Norwegian to English and edited for clarity by the first author.

### 2.5 Ethical considerations

This study was approved by The Regional Ethics Committee (REK Case number 175774) and Sikt - Norwegian Agency for Shared Services in Education and Research (Case number 248550). The principles of informed consent, confidentiality, and assessment of consequences for participants were observed throughout the study. The Regional Ethics Committee (REK) granted dispensation from non-disclosure regulations for the researcher to perform participant observations in the nursing homes. The use of audio recordings was supervised and registered by the Sikt - Norwegian Agency for Shared Services in Education and Research. All participants received written and oral information, were assured of confidentiality, and were informed that they could withdraw from the study at any time. To ensure that information about the study also reached visitors of the nursing home units, a poster with information of the study, a photograph of the researcher, and the researcher's contact information was placed in strategic places in each participating unit.

## 3 Results

Influence and actions that can be interpreted as nudging were regularly observed in the participating nursing homes. While this study focused on caregivers nudging the residents, nudging was also used to influence caregivers’ actions. This is demonstrated in the following field notes example:

***Unit 1 Field notes:****While making the residents’ bed, the caregiver takes equipment meant to protect the resident from developing pressure ulcers from a chair next to the resident's bed and puts it in the bed under the duvet. I ask the caregiver to explain what she is doing and her reasoning. The caregiver explains, ‘I noticed that the resident did not use this last night. I am putting it here in the bed so that the caregiver on the evening shift sees it and remembers to use it.’*

As stated, the caregiver deliberately modified the environment to influence the actions of the caregiver on the evening shift. Another example of nudging aimed at influencing caregivers was the placement of hand disinfectants and face masks at strategic places in the unit to enhance compliance to the new hygiene practices during the coronavirus disease 2019 (COVID-19).

The influence on residents’ decisions, actions, and behaviors interpreted as nudging in this study is described in two categories: Nudging residents via modification of the physical surroundings, and modification of interactions and verbal information.

### 3.1 Nudging as modification of physical surroundings

The fieldwork revealed that modification of physical surroundings was an overarching and thought through strategy to nudge residents’ behaviors towards the units’ goals. In nursing home unit 1, a regular long-term unit, the goal was to make residents feel at home and experience well-being and belonging. In this unit, residents’ rooms displayed their private belongings to enhance their feelings of being safe and at home.

**Unit 1 field notes*:****The room is decorated with the resident's private belongings. There are photographs, flowers, green plants, Christmas decorations, elves, tablecloths on the tables, and the TV stands on an old chest of drawers.*

In the dementia unit (Unit 2), the goal was to maintain calmness and reduce agitation; hence, the unit was designed for a soothing environment. The small, secure unit allowed residents to move freely without the temptation to exit. Nature landscapes, familiar pictures, and calming music contributed to a serene atmosphere. Conversely, in the short-term unit (Unit 3), the aim was rehabilitation for a potential return home. Residents in this unit were considered patients, and personal decorations were prohibited. However, motivational pictures decorated the walls conveying messages of hope, motivation, and personal responsibility for their own well-being.

**Unit 3 field notes:***(The first day in unit 3) The dining hall is extremely impersonal. It is in significant contrast to the seating groups in the dementia unit. The dementia unit felt calm, peaceful, and cozy. Although it was minimally decorated, it was still a pleasant and enjoyable space. This room feels cold and barren. The dementia unit has a garden view. Here, there is only a view of corridors and other rooms, no outlooks onto nature. It feels like an institution, not a place to relax.*

All units featured distinctly marked room doors, visible clocks, and facilities promoting independence such as handrails, raised chairs, and toilets. Meals designed to improve nutrition were sometimes prepared in the unit to enhance residents’ appetites through smell and anticipation. Tables were set elegantly using a black placemat in the dementia unit for better visibility.

**Unit 3 field notes**: The caregiver transfers the sandwiches to the residents’ plates with a spatula. Caregiver X explains that typically they would serve the food by putting bread and cold cuts on the table for the residents to make their own sandwiches so that they can maintain or practice the necessary skills for preparing their own food. Serving premade sandwiches is due to COVID-19 restrictions.

Caregivers enriched the sandwiches with nutritious, high protein fillings to nudge residents towards a larger nutrition intake. Caregivers explained that they endeavored to make mealtimes a pleasant social experience for the residents, regularly considering and discussing residents’ seating at the table.

**Unit 2 A1 interview**: *After we started decorating the food a little extra and changed the way we serve the meals, they (the residents) eat more.*

In various care situations, caregivers utilize environmental modifications to influence resident behavior. Simple interventions, such as providing a wet cloth or a toothbrush, encouraged self-care. Music was used to influence residents’ mood, and items like duvets, doors, or chairs were strategically placed and moved to guide residents in specific directions.

### 3.2 Nudging as information and interaction

Both the fieldwork and the interviews highlighted that experienced caregivers adapt their approach to improve resident compliance and increase the likelihood of achieving residents’ goals, as described by the following physiotherapist:

**Unit 3 A9 interview**: *I can say something like; “Let us go see the guinea pigs,” and then when we are there: “Now that we're here, maybe you can sit 10 times in this chair…”. In a way I try to entice and trick a little more out of each situation with the residents: “We can take a trip up the stairs too, now that we are here….”*

Caregivers nudged residents toward independence by strategically waiting for residents to take initiative. Caregivers limited choices, framed information, and organized alternatives to encourage optimal decision-making.

**Unit 3 fieldnotes***: The occupational therapist explained how she uncovered residents’ preferences for coffee or tea before their first meeting. When later asking residents about their choice of beverage, she strategically placed the preferred option at the end of the sentence, facilitating the resident's response to make it easier for the resident to provide the right answer.*

Caregivers refrained from posing open questions, considering them more challenging and potentially more confusing for residents. They explained that they used this approach to discourage residents from making unsuitable choices and prevent confusion. Caregivers explained and demonstrated how they deliberately employed positive language, tone, and body language to foster cooperation and compliance from residents.

**Unit 2 fieldnotes:***Nurse X explained that to prevent agitation when approaching residents, I must first establish contact with the resident. For example, I could do so by saying the resident's name. You must not place yourself in front of the resident, but slightly at an angle, so that the resident does not feel confronted. The residents should always feel that they have a way out of the situation. Do not use the words “no” or “don't” or any other negative words. Negative words might trigger the residents. Always be positive. For instance, you can say “Come with me” instead of “Don't go there.” You must always be calm and relaxed when you are with the residents.*

In this example, the caregiver explained her approach towards residents with severe dementia, meticulously considering every aspect of her interaction, including her positioning in the room, body language, tone of voice, and the use of positive words, to prevent agitation and foster compliance.

### 3.3 Nudging as an ambiguous intervention

When interviewing caregivers, they first substantially advocated for residents’ rights to choose, including the residents’ rights to refuse care. Caregivers stated that if residents did not want to do something, they respected that decision. When later asked in the interview to recount instances where they felt they had succeeded as caregivers, the respondents typically described scenarios where they had effectively made residents accept care that was initially refused. For example:

**Unit 1 A2 interview***: We had this patient who never wanted to shower. Usually I would ask her “Do you want to go to the bathroom?” and then I would ask “Do you want to shower?” and she would always say no. But this time I just turned on the water without asking her, and I started showering her feet, and then she said, “Oh, that's lovely” and then, little-by-little I could give her a shower. It sometimes works when you just take it little by little, in very small steps.*

When caregivers were asked to reflect on the situations where residents refused care and how they handled these incidents, they began to express conflicting thoughts about the legitimacy of their approaches. For example:

**Unit 1 A4 interview:***We do motivate and coax residents. We try to make residents participate and motivate them… It might border on manipulation sometimes… It's almost the same really…. But we want the best for the residents. We do it for the residents….. But, it is kind of on the border of manipulating residents sometimes.*

This ambiguity was also evident in the language used by caregivers when discussing their approaches to residents in different situations and the strategies used to influence residents. Terms such as “to guide,” “to assist,” and “to help” suggested a positive attitude towards this influence. A more neutral position was indicated using terms like “to motivate” or “to lead.” However, terms like “to push,” “to use tricks,” “to pry,” and “to have or use techniques” were interpreted as indicating a negative stance towards the legitimacy of the influence exerted on the residents.

The observations and field notes disclosed that during morning care routines, some caregivers frequently asked residents about their preferences, while others rarely asked them.

**Unit 1 fieldnotes***They read between the lines, and engage in casual conversation while providing care. They seem confident and predictable. They do not stop to ask the residents what? or how? I have never seen them skipping important tasks. They respect residents’ choices when appropriate but insist on necessary hygiene practices: “No, we're going to do that too,” is a typical answer.*

In the care interactions where caregivers frequently asked residents about preferences, the verbal interactions primarily centered on task performance. This approach also led to extended and sometimes exhausting routines for the residents, limiting personal conversations. In contrast, the caregivers who did not ask the residents about their preferences employed subtle non-verbal cues to guide residents’ choices and actions, while simultaneously engaging them in meaningful conversations about their day, relatives, and other personal matters. Repeated observations revealed a notable consistency in the routine with different residents, which appeared more personalized through the personal conversations while performing care routines. These practices resulted in more efficient routines, leaving residents less fatigued and providing more stimulating personal conversations. One of the residents commented:

**Unit 1 fieldnotes**: *One resident told me that he preferred to be cared for by the older caregivers. I asked him why he preferred these, and he replied that when they helped him, it was as if the care routine just happened naturally, all by itself. He confirmed that he was talking of the same caregivers I was thinking about*.

The proactive approach conveyed by these experienced caregivers suggested that they intervened before residents made unwanted choices.

**Unit 1 fieldnotes***: This resident typically says she will brush her teeth later or after breakfast. Today, before the resident could say something, the caregiver gave the resident the toothbrush and said, “This is important,” with a firm voice. The resident took the toothbrush and started brushing her teeth. On previous days, caregivers intervened only after the resident expressed that she wanted to postpone. Today was the first time the resident brushed her teeth properly since I came.*

This example shows how the experienced caregiver's proactive approach and leadership during the morning care routine ensured that the resident maintained good oral hygiene, in contrast to the caregivers who focused on maintaining residents’ autonomy. This indicates a hierarchical power dynamic in the caregiver-resident relationship.

During interviews, caregivers shared that they engaged in exchanges on effective strategies for working with residents, learning from each other's experiences. One nurse detailed:

***Unit 1 A5 interview****: There are many who have worked here for a very long time, and they know the residents and what works, and it's nice when they share their knowledge.*

The field notes also indicate that experienced caregivers asked fewer questions of residents and employed this form of influence more frequently than less experienced caregivers.

## 4 Discussion

This study's data revealed that influence aligning with the concept and the phenomenon of nudging was frequently applied in the nursing home. This form of influence was directed towards all individuals, including residents, employees, and visitors. Observations showed that caregivers were nudging residents and each other.

We found that the physical surroundings in the nursing home and information and interactions with residents were deliberately adapted to influence choices and behaviors to align with the concept of nudging. These deliberate modifications, aimed at guiding residents’ behavior, were evident in data from both observations and interviews. The modifications were implemented before residents made their choices, subtly suggesting one action or choice over another without openly advocating for a specific choice; therefore, these aligned with the concept of nudging (Thaler and Sunstein, 2008). We also found that the options suggested by these adjustments likely could be resisted if residents strongly preferred not to conform, further aligning these practices with the concept of nudging (Thaler and Sunstein, 2008). This indicated that nudging as a choice and behavior-modifying intervention was a regular practice in the nursing home, as previously asserted by Hanna and Grill (2018). Yet are these instances of nudging observed in our study really nudging?

Though examples in the findings align with the criteria of nudging and commonly used nudging techniques, like timing and framing information (Thaler and Sunstein, 2008), our findings also extend beyond nudging. When participants described their experiences influencing residents, they discussed methods beyond the typical scope of nudging. For example, they talked about their strategies to influence residents' decisions and behavior, which can sometimes involve degrees of pressure, manipulation, or rational persuasion (Blumenthal-Barby, 2012)—nudging is only one small category of such strategies, and is often regarded manipulation (Holm, 2019). Discerning between nudging and other types of influence is difficult, especially since the concept of nudging is still new in the nursing home context. Caregivers likely employ several methods to influence residents at the same time with the aim of giving residents quality care.

Notably, systematic and deliberate modifications of the physical environment aimed to create a homely feeling to foster well-being and a sense of belonging in the long-term ward, maintain calmness, and reduce agitation in the dementia unit, and promote rehabilitation and recovery in the short-term and rehabilitation units. Consequently, the goal of nudging was to encourage residents to align with institutional values and objectives. Each unit was designed to enhance residents’ independence and self-management abilities, featuring distinctly marked room doors, visible clocks, handrails, raised chairs and toilets, and black placemats with white plates to enhance the visibility of food during meals. Furthermore, caregivers used nudging to improve residents’ fulfilment of fundamental needs such as nutritional intake, activity, and hygiene. Similarly, Wahlroos et al. (2023) found that the physical environment of long-term care settings contributes to maintaining self-management and independence in older people. Bourdon et al. (2022) also stated that the importance of physical modifications of nursing home environments is supported by numerous studies, concluding that environmental features have a positive influence on the health of nursing home residents. According to Mortensen et al. (2019), nudging is essential for nurses to facilitate patient movement towards shared goals, where nurses act as leaders, bearing the responsibility of aligning their knowledge of nursing and the institution with the patients’ goals and values to ensure quality care. This implies that nudging, as a manifestation of this power, is a critical tool in achieving institutional goals related to providing quality care for residents in nursing homes.

Critics of nudging claim that nudging is ethically problematic in the clinical context because it is manipulative and undermines autonomy (Holm, 2019), consequently undermining residents’ dignity. However, this study's data makes it challenging to perceive the observed occurrences of nudging as ethically problematic or as undermining resident autonomy and dignity. Adhering to social values and norms is vital for maintaining social dignity (Killmister, 2020), and independence, self-care and cleanliness are also crucial norms granting dignity. From this perspective, these instances of nudging are beneficial and supportive, as they align with the nursing home unit's aims and the reasons for the residents’ stay in the care facility, and promotes their wellbeing. Notably, actions that align with the concepts of nudging and soft paternalism are also understood as supportive of autonomy in other studies (Cameron et al., 2020; Van Loon et al., 2023; van der Weide et al., 2023). This indicates that the perception and interpretation of such actions can vary widely among researchers, shedding light on the need for a careful and nuanced approach when implementing these concepts in healthcare settings.

Although considered paternalistic, nudging does not necessarily disregard autonomy or imply disrespect. Paternalism can also communicate recognition of the individuals’ worth and need for protection (Killmister, 2018). Moreover, not all situations involving choices are situations requiring autonomy because they may not involve decisions pertaining to self-governance driven by second-order values and desires (Killmister, 2018). Second-order values and desires refer to those values and desires which individuals aspire to possess. The long-standing values that guide our actions towards long-term goals contrast with first-order values that direct our actions towards short-term goals and immediate desires (Killmister, 2018). Within the nursing homes, nudging appears to primarily occur in circumstances typically not considered as autonomy situations. However, the question arises as to whether adherence to the nursing home unit's values and goals are primarily intended to benefit the institution's operations or the residents themselves? The ethical implications of nudging and soft paternalism should always be considered, with a focus on ultimately promoting resident autonomy and well-being. It is crucial to remember that residents have the right to choose how their fundamental needs are met while in the nursing home, particularly when this choice involves residents’ values.

The caregivers in this study emphasized their commitment to respecting residents’ choices, asserting that they would respect a resident's decision to refuse certain actions. This strong emphasis on residents’ rights to choose, especially at the beginning of the interviews, is understandable given the focus on enhancing residents’ autonomy to maintain their dignity (Moilanen et al., 2021; Moilanen et al., 2022). However, caregivers also expressed a sense of accomplishment when they could overcome residents’ initial refusal and successfully deliver necessary care, especially to those residents known for refusing care. This complexity underscores the dilemma faced by caregivers in nursing homes: the need to reconcile the demand for autonomy with their responsibility toward care for the residents. The feelings of achievement and accomplishment expressed by caregivers suggest successful fulfilment of their care responsibilities toward the residents. However, reconciling the demand for autonomy with the caring responsibility is challenging. The boundaries between care and restraint become blurred when caregivers struggle between their moral ideals about respecting the residents’ autonomy and free will and the realities they face in interacting with them (Jakobsen and Sørlie, 2010; Øye and Jacobsen, 2020). Studies show that these dilemmas cause feelings of inadequacy and cognitive dissonance in caregivers (Jakobsen and Sørlie, 2010; Mortensen et al., 2022).

The language that caregivers use when discussing their approaches and influence on residents reflects this ambiguity. However, the variation in wording may also suggest a range in the intensity of caregivers’ influence on residents, from subtle guidance to more assertive direction, and its legitimacy depending on the circumstances. This is similar to the differentiation between soft and strong paternalism based on how easy the influence is to resist and whether it benefits the person being influenced (Killmister, 2018; Thaler and Sunstein, 2008). When caregivers use phrases such as “to guide,” “to assist,” “to help,” and “to motivate,” it implies a positive attitude towards gentle influence that residents can easily resist in situations wherein the influence is considered benign. Conversely, terms like “to lead,” “to push,” “to use tricks,” “to pry,” and “to have or use techniques” suggest stronger and potentially more controversial influence.

Autonomy and paternalism, although often discussed as binary or absolute terms, exist on a spectrum in practice, with their applications varying considerably based on the circumstances. The shaping of this spectrum is influenced by several factors, including the methods used to exert the paternalistic influence and the intricacy of the given situation. The complexity of the situation encapsulates, not only the possible harm an undesired action might pose, but also whether the action aligns with the resident's broader values and wishes (Killmister, 2018), and if the resident will likely appreciate the action thereafter. This indicates that caregivers, in practice, navigate a complex array of considerations when exerting paternalistic influence over residents, and their perception of this influence can shift based on these factors.

This interpretation suggests that caregivers perceive paternalistic influences on a spectrum from very gentle to assertive influence or coercion. Gentle influence, akin to the nudging described in the findings, is perceived as legitimate by the caregivers, whereas stronger influence is perceived as more questionable, requiring stronger justification. This aligns with the notion of nudging as soft paternalism that is easy to resist (Thaler and Sunstein, 2008). To the caregivers, nudging may represent the most subtle form of influence, closely associated with guiding, assisting, and helping. As caregivers exert more than a nudge, venturing into argumentation and coaxing, their perception of the influence becomes more negative.

Interestingly, caregivers who constantly asked for residents’ preferences during morning care routines often ended up with longer, task-focused routines, leaving the residents exhausted. Conversely, caregivers who subtly guided residents through non-verbal cues managed to create more efficient routines that were less draining for the residents. These caregivers engaged in personal conversations during the care situation, offering a more personal and enriching experience for the residents. Experienced caregivers tended to take this proactive approach, guiding the resident through the morning routine, whereas less experienced caregivers focused on the residents’ preferences. Initially, we interpreted this as experienced caregivers being more familiar with the residents, thereby being able to tailor care to their preferences without needing to ask numerous questions. However, further observations showed that these experienced caregivers performed the care routine in similar ways for different residents. This suggests that the morning routine was primarily dictated by the caregiver rather than the resident. This finding is intriguing because autonomy has traditionally been closely linked to respect and the preservation of dignity. Previous studies suggest that residents perceive that they have autonomy when they can make choices about their daily lives and achieving autonomy is associated with promoting both health and quality of life (Moilanen et al., 2021; Van Loon et al., 2023). Caregivers stimulating residents to make choices about care is typically interpreted as strengthening residents’ autonomy similar to Van Loon et al. (2023) interpretation. This carries an inherent presumption that more choices equal more autonomy, and more autonomy reflects better care quality and dignity. However, our findings indicate that more opportunities for choice during morning care routines may not improve the quality of care or enhance dignity. Furthermore, there were indications of qualitative differences in the methods of influencing residents’ choices.

These observations may have been influenced by the presence of the researcher. The less experienced caregivers, possibly aiming to demonstrate their respect for residents’ autonomy, may have been more affected by the researcher's presence. In contrast, the more experienced caregivers were likely less influenced by this presence. However, caregivers expressing in interviews that they learned and exchanged effective strategies for working with residents strengthens the assumption that experienced caregivers had a larger repertoire of skills for leading and guiding residents. The residents’ comments of preferring these caregivers also indicates that this difference is genuine. These findings suggest, contrary to previous understanding, that an excessive focus on residents’ wishes could negatively affect the quality of interaction between caregivers and residents. Using nudging to guide residents through the care routine can improve the achievement of care goals, such as maintaining good hygiene, and lead to better interactions between caregivers and residents.

### 4.1 Strengths and limitations

The fieldwork for this study was performed during stringent COVID-19 restrictions in nursing homes. The use of face masks to limit the spread of the virus impacted communication during the participant observations. Specifically, the facemasks obscured the faces of the participating caregivers, making it difficult for residents to see and read the caregivers' facial expressions during care activities and interactions. To compensate for their limited ability to communicate using facial expressions, caregivers might have given more explicit signals using words and body language. Notably, residents commented that they adapted to the lack of facial expressions. This limitation was not present during interviews as the researcher and the participants were able to maintain the necessary distancing; hence, face masks were not required.

The combination of participant observation and interviews at the nursing home strengthened the quality of the interviews, providing a common reference framework for the interviewer and participants before the interviews. This also enabled the interviewer to ask better questions and validate some of the early interpretations of the observations. This was also a strength during the analysis and interpretation of the interviews and field notes.

While the results of this study provide valuable insights, they are derived from a limited sample in a specific context. The unique characteristics and conditions of the nursing homes studied may have influenced the findings. These findings might not apply universally to all nursing homes or healthcare institutions. The inherent subjectivity and complexity of qualitative research also limit the extent to which these findings can be generalized.

Despite these limitations, this study provides valuable contributions to our understanding of the use of nudging in nursing homes and offers a foundation for subsequent investigations. Additional research is required to confirm and expand upon the findings. Specifically, future research should focus on developing a better understanding of the ethical aspects of nudging from residents' viewpoints, assessing nudging's adaptability across different care settings, and identifying training methods for caregivers.

## 5 Conclusions

This study revealed that nudging, prevalent in nursing homes, is used to guide residents’ choices and behaviors to promote their well-being and uphold social norms. The physical surroundings and interactions with residents are deliberately adapted to encourage choices that align with nursing home goals. While nudging primarily occurs in situations not typically involving autonomy, it often guides residents towards actions consistent with their personal values and goals. The study also uncovered the complex considerations caregivers navigate when exerting influence over residents. This influence shows a spectrum from subtle nudging to more assertive argumentation and coaxing. Nudging is perceived as the most subtle form of influence, closely associated with guiding, assisting, and helping. The study also found that excessive focus on residents’ wishes can sometimes negatively impact caregiver-resident interaction. Experienced caregivers use nudging effectively to guide care routines, resulting in efficient care and better interactions than those who constantly ask for residents’ preferences. This study acknowledged the ethical concerns regarding nudging potentially infringing on residents’ autonomy; however, in the nursing home context, nudging is observed to protect and support residents’ dignity. Finally, this study underscored the importance of using nudging responsibly and ethically to enhance the quality of care and to promote residents’ well-being in nursing homes. Ultimately, this study provides valuable insights into the role and impact of nudging in nursing homes. It shows that nudging can positively shape resident behavior and choice, with potential benefits for staff training and care quality improvement. These findings should also reassure caregivers that, when used responsibly, nudging can be a beneficial tool in their interaction with residents, without any associated guilt or negative connotations. However, these findings are contextual, and their applicability may vary based on different nursing home environments and populations.

## CRediT authorship contribution statement

**Anne Helene Mortensen:** Writing – original draft, Project administration, Methodology, Investigation, Formal analysis, Conceptualization. **Dagfinn Nåden:** Writing – review & editing, Validation, Supervision, Methodology, Formal analysis, Conceptualization. **Dag Karterud:** Writing – review & editing, Validation, Supervision, Methodology, Formal analysis. **Vibeke Lohne:** Writing – review & editing, Validation, Supervision, Project administration, Methodology, Formal analysis, Conceptualization.

## Declaration of competing interest

The authors declare that they have no known competing financial interests or personal relationships that could have appeared to influence the work reported in this paper.

## Appendix Supplementary materials

Image, application 1

Image, application 2
