# Corrigendum to “Nudging in the nursing home: A qualitative interpretive study” [International Journal of Nursing Studies Advances 8 (2025) 100287]

**DOI:** 10.1016/j.ijnsa.2025.100314

**Published:** 2025-02-21

**Authors:** Anne Helene Mortensen, Dagfinn Nåden, Dag Karterud, Vibeke Lohne

**Affiliations:** Faculty of Health Sciences, Department of Nursing and Health Promotion, OsloMet–Oslo Metropolitan University, Oslo, Norway

The authors regret that the abstract incorrectly states that the study was conducted between December 7th, 2019, and July 2nd, 2020. The correct period is between December 7th, 2020, and July 2nd, 2021.

The authors would like to apologise for any inconvenience caused.

