# Supplementary material for: Nudging in the nursing home: A qualitative interpretive study
Source: Int J Nurs Stud Adv. 2024 Dec 29;8:100287. doi: 10.1016/j.ijnsa.2024.100287 (PMC11762191; doi:10.1016/j.ijnsa.2024.100287)
Supplement: Supplementary file 1 [file mmc1.docx]

Data Availability Statement

The raw/processed data required to reproduce the findings in this manuscript cannot be shared due to legal and ethical reasons.
