# Supplementary material for: Nudging in the nursing home: A qualitative interpretive study
Source: Int J Nurs Stud Adv. 2024 Dec 29;8:100287. doi: 10.1016/j.ijnsa.2024.100287 (PMC11762191; doi:10.1016/j.ijnsa.2024.100287)
Supplement: Supplementary file 2 [file mmc2.docx]

**Interview guide nudging in nursing home context**

(Translated from Norwegian)

Repeat information about interviewer and the study, ask if it is ok to turn on the tape recorder.

**Background information:**

- Age, profession, how long you have worked at the nursing home, previous experience.
- How many residents do you have? How many employees? Care assistants? Nurses? Are there others assigned to this unit?

**What do you do if a resident resists care?**

- Do you have ways of preventing this from happening?
- Please tell me more

**Questions about goals, values and aims**

- What do you think is important in your work here? Do you have common goals you work from?
- What do you do to achieve these goals?
- What about the residents, do they have goals or a purpose or something they are trying to achieve?
- What do you do to help them achieve this?

**Have you experienced residents where you find it difficult to do a good job?**

- Can you describe a situation that you consider difficult?
- What do you do in such situations?
- How do you experience being in this situation?

**Can you describe a situation that you consider being on the border between voluntariness and pressure/coercion**

- What do you do if a resident resists something you believe is necessary?
- Is motivating residents an issue in the department? How do you work with that?

**Have you experienced situations where you are unsure if what you are doing is okay? (in relation to coercion/voluntariness)**

- Please elaborate

**Can you tell me a story about once you felt successful at work or a situation where you felt you did particularly well**?

- What contributed to your success in this situation?
- Please elaborate

**What do you find challenging/difficult working here?**

- Please elaborate

**According to theory about decision making, you can influence someone’s decisions by means of rational arguments, by giving incentives or rewards, or by portraying one alternative as more tempting than the other alternative.**

- What do you think about this?
- Do you ever use any of this with residents?

**What do you do if a resident is known to resist care?** (repeat topic from beginning)

- Do you have ways of preventing this from happening?
- Please tell me more

**Have you ever thought that residents have been tricked or manipulated?**

- Please elaborate.
- Can you tell me where the boundary between motivation, coaxing and coercion goes?

**Follow up questions trough out the interview:**

- Can you tell a little more about that? Do you have examples?
- How do you proceed then?

**Nearing the end:**

Repeat my understanding, ask if I understand them correctly. Give possibility of elaborations

- Is there anything else you want to tell me?
- Is there a question you have missed?

**Thank you for your participation!**
